# Study retention prediction with AI

**DOI:** 10.1192/j.eurpsy.2021.385

**Published:** 2021-08-13

**Authors:** A. Mereu

**Affiliations:** Research performed independently, Cagliari, Italy

**Keywords:** traits, retention, Artificial Intelligence, Personality

## Abstract

**Introduction:**

Openness, conscientiousness, extroversion, agreeableness and neuroticism are dimensional personality traits known as the Big Five. Study attrition is a common but often hard to anticipate problem. Artificial intelligence (AI) could examine both fronts to mitigate the unpredictability of the latter.

**Objectives:**

To investigate whether AI could predict study attrition employing personality traits scores.

**Methods:**

Data from 2,697 questionnaires were analysed using an AI. The short form of the International Personality Item Pool was used to assess the Big Five personality traits on the first of three planned waves. The personality traits scores were employed to predict the missing of at least one wave. Overall attrition was 17.6%. The AI was conservatively tuned to minimize the negative likelihood ratio when confronting predicted and real attrition. The free and open source programming language R was used for all the analyses. Dataset source: Hansson, Isabelle; Berg, Anne Ingeborg; Thorvaldsson, Valgeir (2018), “Can personality predict longitudinal study attrition? Evidence from a population-based sample of older adults”, Mendeley Data, V1, doi: 10.17632/g3jx8zt2t9.1

**Results:**

Predictions obtained a negative likelihood ratio of 0.333 and a negative predictive value of 0.933. The results were indicative of fair performance.

**Conclusions:**

AI might be useful to predict study retention. Furthermore, the results of this study might indicate a moderate effect of the Big Five on the probability of study retention. Finally, the AI used in this study is freely available, allowing anyone to experiment.

**Disclosure:**

No significant relationships.

